# Percutaneous Transluminal Septal Myocardial Ablation for Hypertrophic Obstructive Cardiomyopathy Under Extracorporeal Membrane Oxygenation Support

**DOI:** 10.1016/j.jaccas.2020.07.062

**Published:** 2020-10-21

**Authors:** Takahiro Kobayashi, Hiroyuki Takaoka, Haruka Sasaki, Manami Takahashi, Kan Saito, Tomohiko Hayashi, Kwangho Lee, Yoshihide Fujimoto, Masato Yamanouchi, Yoshio Kobayashi

**Affiliations:** a Cardiovascular Medicine, Chiba University Graduate School of Medicine, Chiba, Japan; b Department of Cardiology, Chiba Rosai Hospital, Chiba, Japan

**Keywords:** ablation, acute heart failure, cardiomyopathy, AF, atrial fibrillation, ECMO, extracorporeal membrane oxygenation, HCM, hypertrophic cardiomyopathy, HOCM, hypertrophic obstructive cardiomyopathy, LV, left ventricular, LVOT, left ventricular outflow tract, PTSMA, percutaneous transluminal septal myocardial ablation, TTE, transthoracic echocardiography, VT, ventricular tachycardia

## Abstract

We report the case of a 70-year-old woman with hypertrophic obstructive cardiomyopathy, who was admitted because of severe heart failure and cardiogenic shock and mechanical support requiring extracorporeal membrane oxygenation. She recovered well by percutaneous transluminal septal myocardial ablation under the extracorporeal membrane oxygenation support and was discharged without complications. (**Level of Difficulty: Advanced.**)

## Graphical abstract

**Figure:**
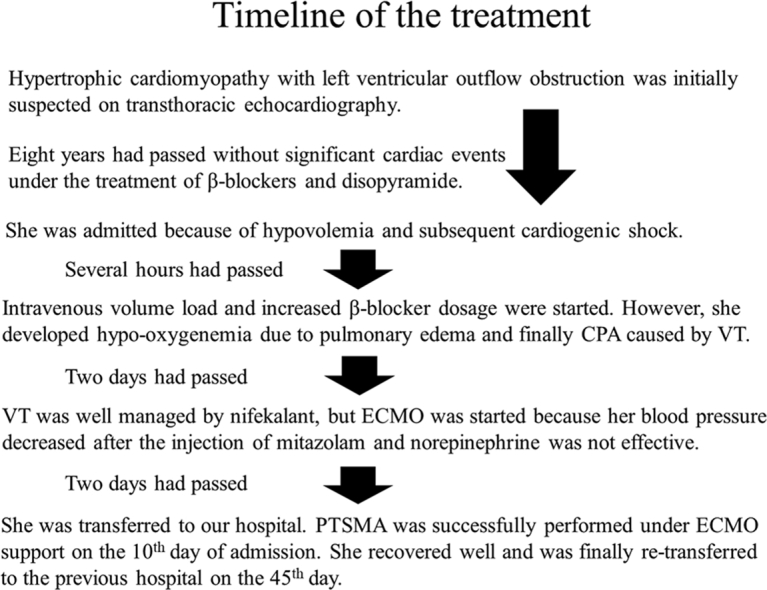

## History of Presentation

**Figure 1:**
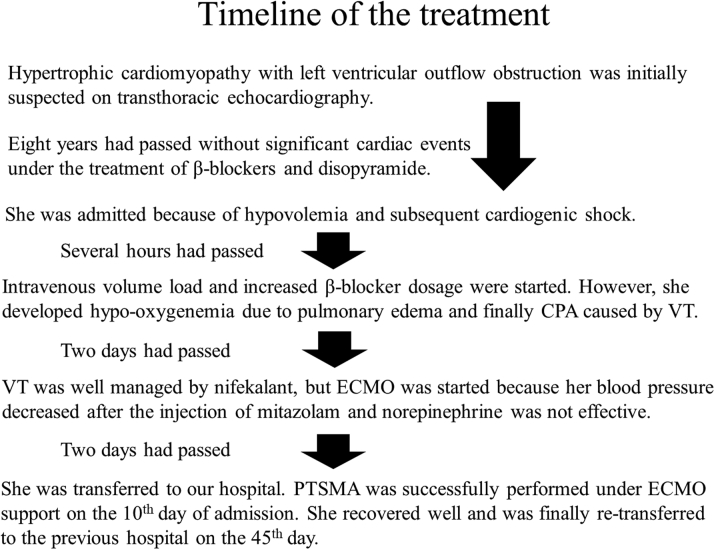
Treatment Timeline CPA = cardiopulmonary arrest; ECMO = extracorporeal membrane oxygenation; PTSMA = percutaneous transluminal septal myocardial ablation; VT = ventricular tachycardia.

A 70-year-old woman with hypertrophic obstructive cardiomyopathy required extracorporeal membrane oxygenation (ECMO) support due to cardiogenic shock and acute heart failure and showed dramatic improvement after a percutaneous transluminal septal myocardial ablation (PTSMA) (Figure 1).

**Learning Objectives**

• To learn how to manage patients with severe heart failure caused by HOCM under ECMO.
• To understand the effects of PTSMA in patients experiencing cardiogenic shock due to HOCM.

The patient became aware of exertion-related chest symptoms almost 9 years ago and presented to another hospital. Transthoracic echocardiography (TTE) revealed a diffuse left ventricular (LV) hypertrophy, except in the posterior wall, and the maximum wall thickness of the LV was 20 mm. The maximum velocity of the left ventricular outflow tract (LVOT) was 503.2 cm/s on Doppler echocardiography, and the pressure gradient of the LVOT was estimated as 101.3 mm Hg. The patient was diagnosed with hypertrophic obstructive cardiomyopathy (HOCM). She was treated with β-blockers and disopyramide. The pressure gradient in the LVOT decreased to 30 mm Hg, and she was well managed without significant cardiac events, except for syncope, which occurred several times over 8 years. Thereafter, she was diagnosed with traumatic subarachnoid hemorrhage caused by syncope, but no remarkable changes on TTE or arrhythmia were detected after the event; she recovered well without neurological sequelae. Two weeks after the event, she was brought to the emergency room due to nausea, dizziness, and chest oppression resulting from intensive work in a hot room. Her blood pressure and maximum LVOT velocity were 67/48 mm Hg and 4.3 m/s on TTE, respectively; therefore, she was diagnosed with cardiogenic shock with hypovolemia and admitted to the hospital. Intravenous volume load was started, and the dosage of the β-blocker was increased because hypovolemic shock was considered to exacerbate LVOT stenosis. Several hours later, she developed hypo-oxygenemia due to pulmonary edema and cardiopulmonary arrest caused by pulseless ventricular tachycardia (VT). After recovery from cardiac arrest by defibrillation, injection of epinephrine, and mechanical ventilation, continuous injection of nifekalant was started to prevent VT. VT was well suppressed and her condition had been stable for almost 2 days. However, immediately after the injection of 4-mg midazolam to suppress unnecessary body movement, her carotid artery pulse could not be palpated and her systolic blood pressure suddenly dropped to nearly 30 mm Hg, without measurable diastolic pressure. Therefore, 0.15 mg of norepinephrine was infused 3 times but was ineffective; hence, 0.5 ㎍/kg/min of norepinephrine was continuously administered. However, her systolic blood pressure remained low, and venoarterial extracorporeal membrane oxygenation (ECMO) was started on the fourth day. On the fifth day, she was transferred to our hospital for more intensive treatment. A venous cannula was placed in the right common femoral vein for extraction, an arterial cannula was placed into the right or left femoral artery for infusion, and the blood flow of ECMO was regulated at 2.1 l/min (the number of rotations was 2,910/min).

## Past Medical History

The patient did not have any other medical history, except hypertrophic cardiomyopathy (HCM).

## Differential Diagnosis

**Figure 2:**
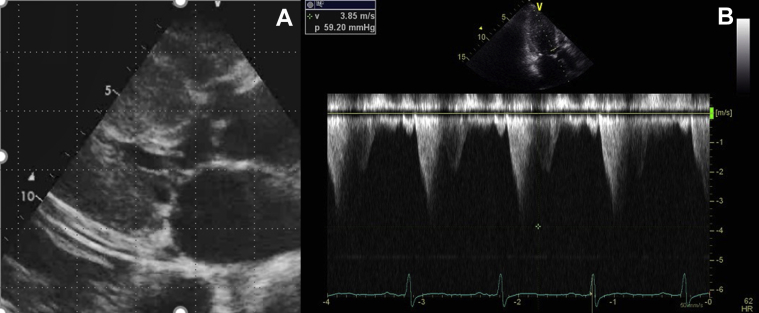
Transthoracic Echocardiography Findings Almost 1 Month Before Admission **(A)** Left ventricular (LV) ejection fraction is approximately 70%, and diffuse severe LV hypertrophy excluding the basal posterior wall is observed. The maximum LV wall thickness was 20 mm. **(B)** Systolic anterior motion of the mitral valve is also observed, and the accelerated blood flow of the LV outflow tract was approximately 3.85 m/s.

Two days after starting ECMO, she was transferred to our hospital. Almost 1 month before the admission, TTE revealed diffuse LV wall thickness of ≥15 mm excluding the posterior segment, with significant LVOT stenosis and a pressure gradient of approximately 60 mm Hg at rest (Figures 2A and 2B), a typical abnormality observed in HCM (Maron type III) (1). Her grandchild had been diagnosed with HCM.

She did not have other cardiac or systemic disease that could account for the magnitude of hypertrophy; therefore, she was finally diagnosed with HCM (2,3).

## Investigations

Results of medical examinations performed after the transfer to our hospital were as follows: blood pressure 108/74 mm Hg, heart rate 122 beats/min, body temperature 35.5°C, and B-type natriuretic peptide level 1,246.4 pg/ml.

**Figure 3:**
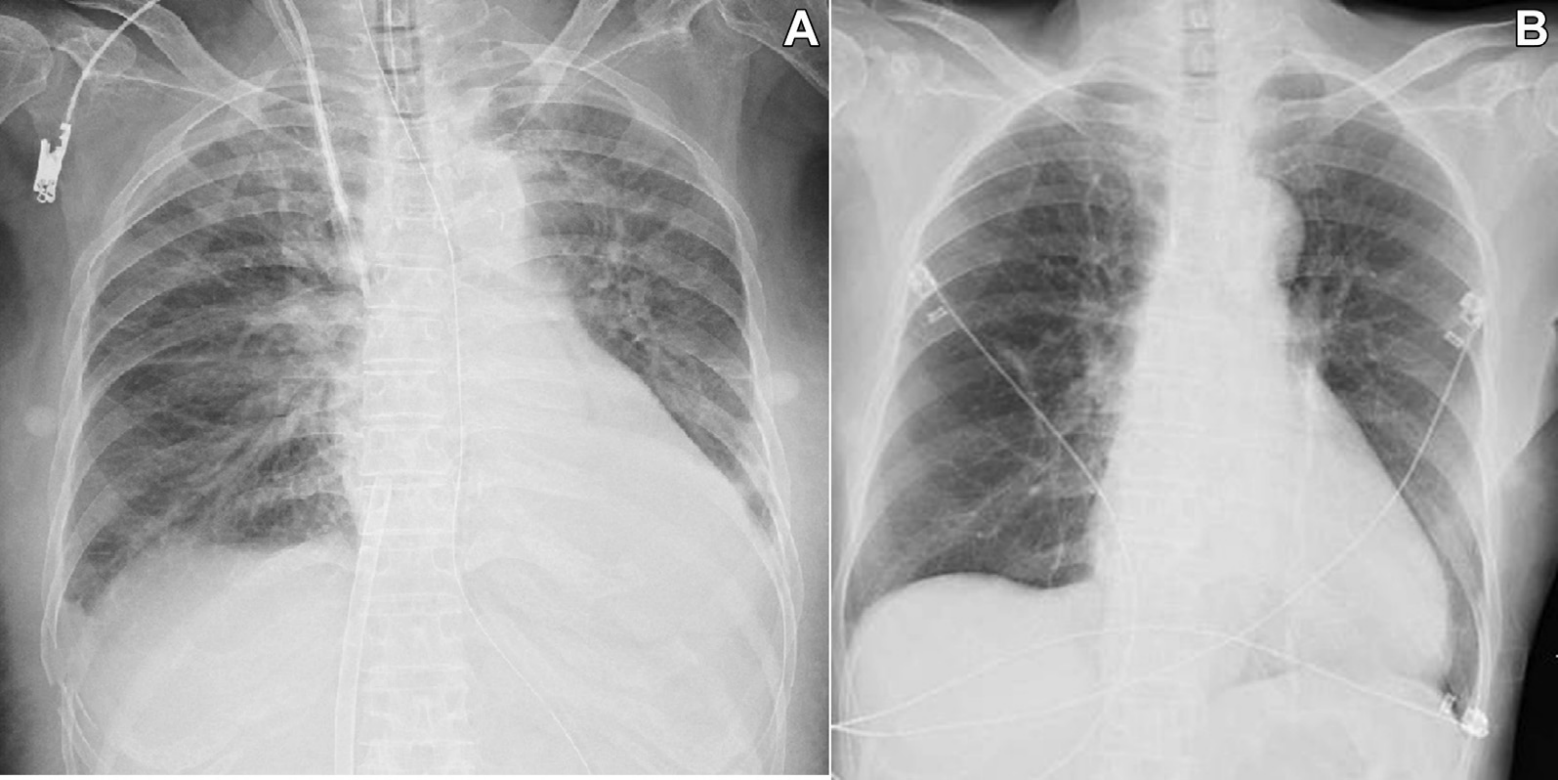
Chest X-Ray Films Before and After Percutaneous Transluminal Septal Myocardial Ablation **(A)** Chest x-ray film showing bilateral pulmonary congestion and pleural effusion. The cardiothoracic ratio is approximately 60%. **(B)** Bilateral pulmonary congestion and pleural effusion are no longer observed after percutaneous septal myocardial ablation.

**Figure 4:**
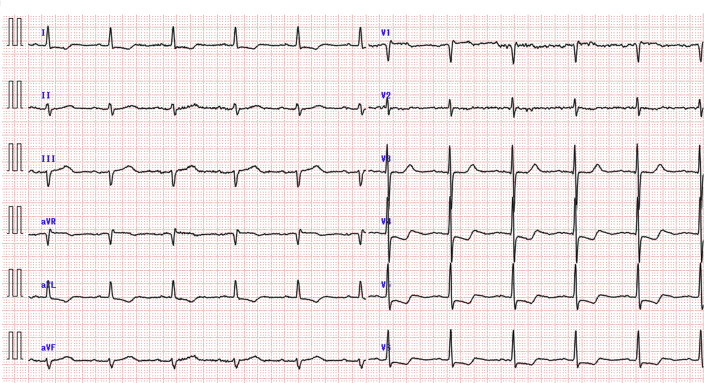
Initial Electrocardiogram After Hospital Transfer Electrocardiogram showing a normal sinus rhythm and ST-segment depression in leads V_3_ to V_6_, aVL, and I.

Chest x-ray film showed bilateral pulmonary congestion and pleural effusion, and the cardiothoracic ratio was 60% (Figure 3A). Her electrocardiogram revealed normal sinus rhythm and ST-segment depression in leads V_3_ to V_6_, aVL, and I (Figure 4). TTE revealed preserved LV contraction and accelerated blood flow through the LVOT at approximately 1.3 m/s. When ECMO flow was reduced from 1.8 l/min to 0.7 l/min, the blood flow through the LVOT increased to 2.3 m/s. The size of the left atrium was 39 mm on TTE.

## Management

Continuous intravenous injection of landiolol was started from 3 μg/kg/min and increased to 10 μg/kg/min; oral administration of 300-mg cibenzoline per day was also started (4). After the onset of atrial fibrillation (AF), cardiogenic shock occurred, and the systolic blood pressure decreased to 80 mm Hg; amiodarone was started, which effectively prevented AF.

**Figure 5:**
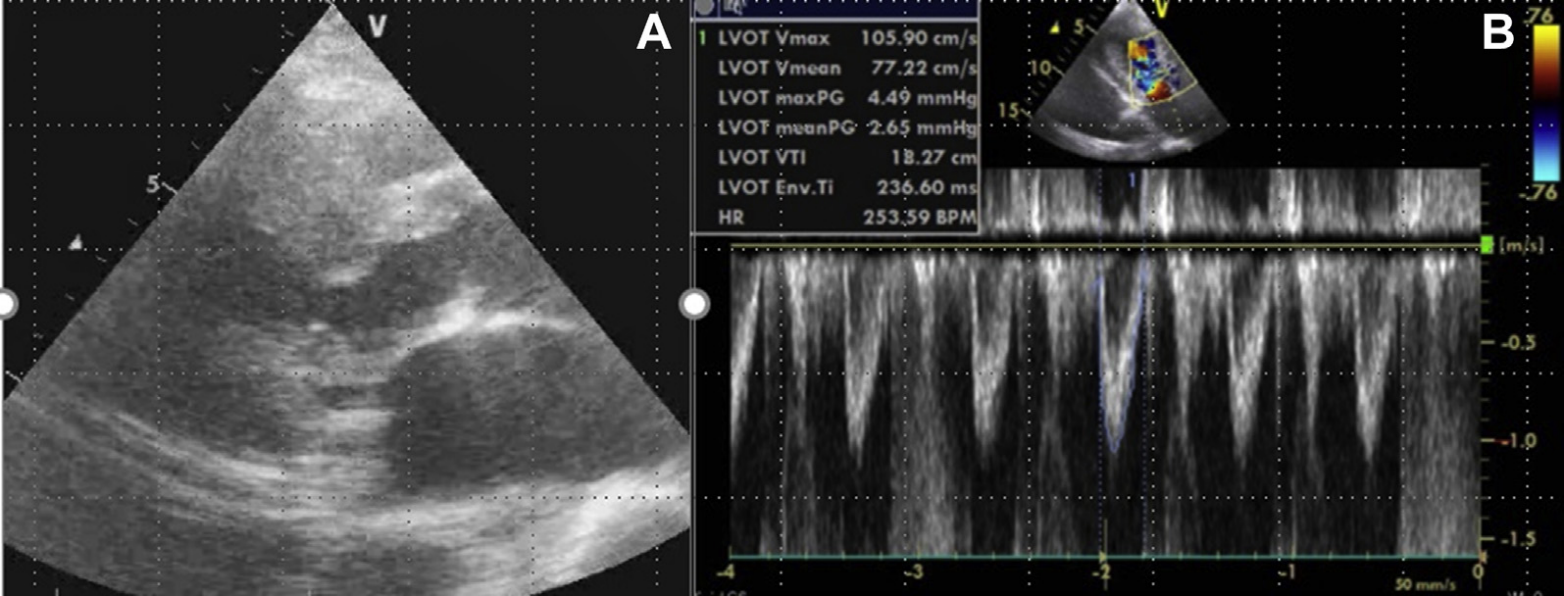
Transthoracic Echocardiography Findings After Percutaneous Transluminal Septal Myocardial Ablation **(A)** Decrease in left ventricular outflow obstruction after percutaneous transcatheter septal myocardial ablation, and **(B)** the accelerated blood flow of the left ventricular outflow tract (LVOT) decreased to approximately 1.1 m/s on transthoracic echocardiography without the support of extracorporeal membrane oxygenation after percutaneous transcatheter septal myocardial ablation.

**Figure 6:**
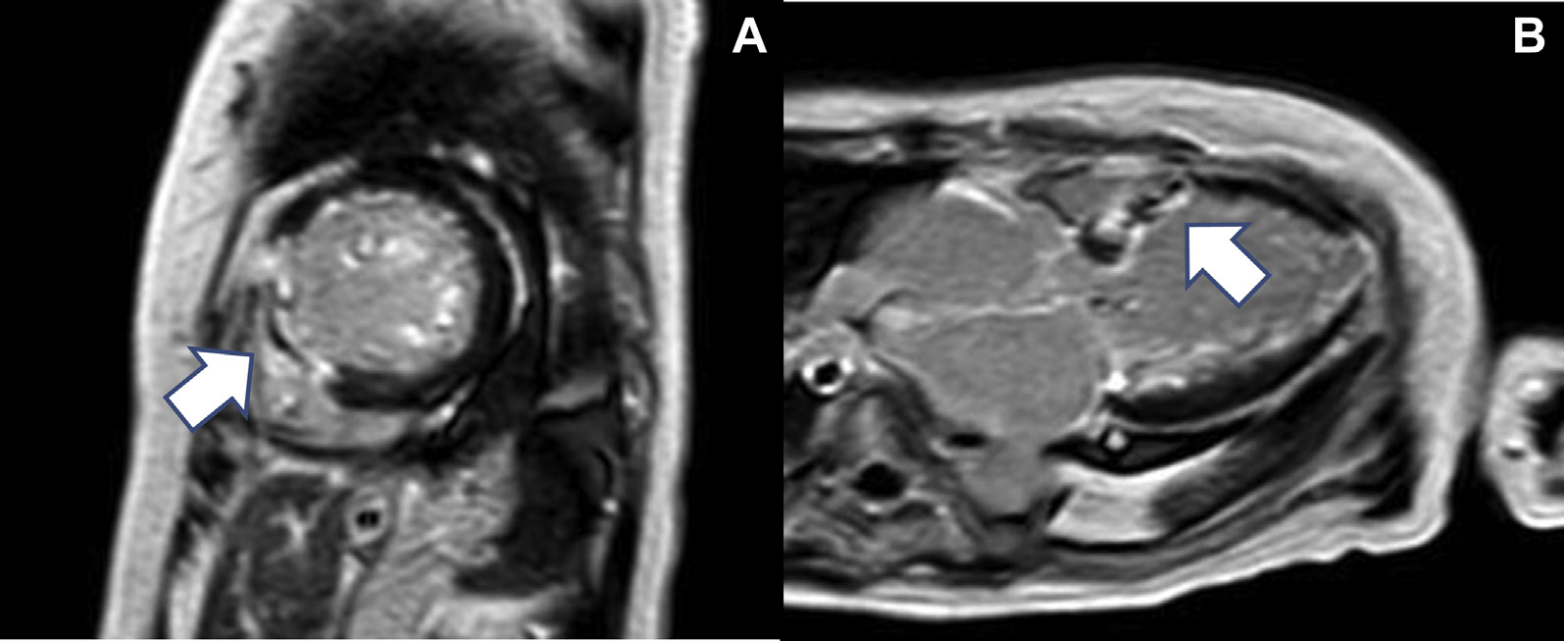
Cardiac Magnetic Resonance Imaging Findings After Percutaneous Transluminal Septal Myocardial Ablation Cardiac magnetic resonance imaging showing late gadolinium enhancement **(white arrows)** in the interventricular septum on **(A)** the short-axis view and **(B)** 3-chamber long-axis view.

Invasive coronary angiography revealed no significant stenosis of the coronary arteries on the eighth day. PTSMA was planned because of the risk of cardiogenic shock caused by the recurrent AF or hypovolemia. On the 10th day, PTSMA was performed, and 1.5 ml of absolute ethanol was injected into the first major septal branch. Her LVOT pressure gradient decreased to 4.5 mm Hg (Figure 5) after the procedure, ECMO support was terminated on the 11th day, and she was extubated next day. Cardiac magnetic resonance imaging was performed on the 22th day, and late gadolinium enhancement was clearly detected in the interventricular septum (Figure 6). Pulmonary congestion finally disappeared on her chest x-ray film on the 25th day (Figure 3B). An implantable cardioverter-defibrillator was implanted for secondary prevention of VT on the 30th day, and she was finally transferred to the previous hospital for rehabilitation on the 45th day of admission.

## Discussion

Patients with symptomatic LVOT obstruction in HCM should be treated initially with nonvasodilating β-blockers titrated to the maximum tolerated dose (2). Class Ia and Class II antiarrhythmic drugs are also recommended to decrease LVOT obstruction and improve symptom (1).

Septal reduction therapy should be considered for patients with drug-resistant HOCM (2), with septal myectomy and PTSMA. They are effective for LVOT obstruction, with similar clinical outcomes (5). This case was regarded as drug-resistant HOCM because the pressure gradient of the LVOT was 30 mm Hg, even with the administration of 2 medicines, and ECMO support was necessary when AF or hypovolemia occurred; therefore, she was considered an appropriate candidate for septal reduction therapy. Minimally invasive treatment was finally selected, considering her age and ECMO treatment. We obtained written informed consent from the patient for the publication of this report.

## Follow-Up

Post-treatment, she was discharged and periodically followed up. Based on our literature review, no other case report described a patient undergoing PTSMA while under ECMO support.

## Conclusions

PTSMA is useful for patients with HOCM, even in those with deteriorated hemodynamic situation under ECMO support.

## Author Relationshp With Industry

This work was partially supported by the TSUCHIYA MEMORIAL MEDICAL FOUNDATION (grant no. J17KF00167). The authors have reported that they have no relationships relevant to the contents of this paper to disclose.
